# Letter to the Editor: First detection of a *Mycobacterium tuberculosis* XDR clinical isolate harbouring an RpoB I491F mutation in a Ukrainian patient treated in Germany, October 2023

**DOI:** 10.2807/1560-7917.ES.2024.29.42.2400665

**Published:** 2024-10-17

**Authors:** Florian Heger, Alexander Indra

**Affiliations:** 1 Austrian Agency for Health and Food Safety (AGES), Vienna, Austria; 2 Paracelsus Medical University, Salzburg, Austria

**Keywords:** tuberculosis, Austria, I491F, sequencing, antimicrobial resistance, AMR, mutation, Mycobacteriacea

**To the editor:** We read with great interest the article by Friesen et al. [1], which reports the detection of a *Mycobacterium tuberculosis* extensively drug-resistant (XDR) clinical isolate harbouring an RpoB I491F mutation in a Ukrainian patient treated in Germany in October 2023. The authors highlight that this mutation is not identified by any of the rapid diagnostic tests recommended by the World Health Organization (WHO), and is primarily associated with lineage 4 strains in Sub-Saharan Africa. The detection of this mutation in a lineage 2 strain, as in the reported case, raises public health concerns, indicating that the mutation is not limited to lineage 4 strains.

As the Reference Centre for Tuberculosis in Austria, we wish to share a strikingly similar case involving a Chechen patient who had arrived in Austria via Belarus nearly 15 years ago. Initially, phenotypic drug susceptibility testing (DST) for rifampicin was conducted before the WHO updated critical concentrations (CC) for susceptibility testing to 0.5 mg/L [2]. Consequently, the isolate was initially classified as rifampicin-susceptible at a CC of 1.0 mg/L and exhibited no mutations in genotypic DST. 

Over the following years, the patient occasionally returned with culture-positive sputum. In 2018, DST of a recent isolate revealed a new resistance pattern, and whole genome sequencing identified an RpoB I491F mutation. Subsequent resistance testing indicated low-level rifampicin resistance, first at a CC of 0.25 mg/L in 2020, and later at a CC of 0.5 mg/L in 2021. 

In 2022, sequencing additionally identified an Rv0678 139_ins_g mutation. Furthermore, throughout the course of therapy, we observed the development of heteroresistance to linezolid due to a mutation in *rplC* (C154R), which subsequently led to phenotypic resistance against linezolid in 2024. 

In contrast to the isolate in Germany, the *M. tuberculosis* isolate from our patient belonged to lineage 3 (Delhi/CAS). As Friesen et al. concluded, the presence of the I491F mutation in lineages beyond lineage 4 raises serious public health concerns, suggesting the potential for widespread transmission and entrenchment of XDR-TB. Our findings reinforce this argument, demonstrating the I491F mutation in a third independent TB lineage (lineage 3 - Delhi/CAS) in eastern Europe. 

The undetected presence of I491F mutations can lead to inadequate susceptibility testing for rifampicin, potentially driving the dissemination of resistance to other essential MDR-TB treatments, such as bedaquiline, linezolid and clofazimine [3,4]. This raises critical questions about the adequacy of diagnostic approaches that do not incorporate comprehensive molecular and phenotypic methods such as sequencing, along with stringent surveillance and monitoring of drug resistance. The threat of rapid XDR-TB dissemination, even in low-incidence countries such as Germany and Austria, is particularly pressing in light of ongoing migration from high-incidence MDR-TB regions, including Ukraine, Chechnya and Belarus, although lineage 3 (Delhi/CAS) is not the predominant lineage in these areas [5].

Furthermore, molecular diagnostics, particularly sequencing, facilitate the early detection of heteroresistance which can often be overlooked by phenotypic methods alone [4].

For these reasons, implementation of sequencing for TB-positive samples at an early stage of diagnosis should be prioritised. For countries with limited technical and financial resources, it is therefore crucial to improve access to quality TB diagnostic methods, as recommended by the WHO [6]. Approaches on a transnational level may be needed to address these challenges.
